# Exploration of Response Mechanisms in the Gills of Pacific Oyster (*Crassostrea gigas*) to Cadmium Exposure through Integrative Metabolomic and Transcriptomic Analyses

**DOI:** 10.3390/ani14162318

**Published:** 2024-08-09

**Authors:** Luyao Dong, Yanan Sun, Muyang Chu, Yuxin Xie, Pinyi Wang, Bin Li, Zan Li, Xiaohui Xu, Yanwei Feng, Guohua Sun, Zhongping Wang, Cuiju Cui, Weijun Wang, Jianmin Yang

**Affiliations:** 1 School of Fisheries, Ludong University, Yantai 264025, China; dly6568@163.com (L.D.); lizanlxm@163.com (Z.L.);; 2 College of Fisheries and Life Science, Shanghai Ocean University, Shanghai 201306, China; 3 Yantai Kongtong Island Industrial Co., Ltd., Yantai 264000, China; 4 Yantai Haiyu Marine Technology Co., Ltd., Yantai 264000, China

**Keywords:** cadmium stress, *Crassostrea gigas*, metabolomic, transcriptomic, energy metabolism

## Abstract

**Simple Summary:**

With the acceleration of global industrialization, marine heavy metal pollution is becoming increasingly severe, posing significant threats to marine ecosystems and human health. To comprehensively understand the response mechanisms of mollusks to cadmium (Cd) stress, we selected the Pacific oyster (*Crassostrea gigas*) as the research species. We aim to delve into the molecular mechanisms underlying the response of oysters to cadmium exposure. Our results indicate that Cd exposure significantly increases Cd concentration in oyster gill tissues, eliciting strong physiological and metabolic reactions, including enhanced oxidative stress response and disrupted energy metabolism, signaling the oyster’s biological response to acute Cd stress. This study provides new insights into the signal transduction and metabolic pathways involved in the acute biological response of bivalves and other marine organisms to heavy metals.

**Abstract:**

Marine mollusks, including oysters, are highly tolerant to high levels of cadmium (Cd), but the molecular mechanisms underlying their molecular response to acute Cd exposure remain unclear. In this study, the Pacific oyster *Crassostrea gigas* was used as a biological model, exposed to acute Cd stress for 96 h. Transcriptomic analyses of their gills were performed, and metabolomic analyses further validated these results. In our study, a total of 111 differentially expressed metabolites (DEMs) and 2108 differentially expressed genes (DEGs) were identified under acute Cd exposure. Further analyses revealed alterations in key genes and metabolic pathways associated with heavy metal stress response. Cd exposure triggered physiological and metabolic responses in oysters, including enhanced oxidative stress and disturbances in energy metabolism, and these changes revealed the biological response of oysters to acute Cd stress. Moreover, oysters could effectively enhance the tolerance and detoxification ability to acute Cd exposure through activating ABC transporters, enhancing glutathione metabolism and sulfur relay system in gill cells, and regulating energy metabolism. This study reveals the molecular mechanism of acute Cd stress in oysters and explores the molecular mechanism of high tolerance to Cd in oysters by using combined metabolomics and transcriptome analysis.

## 1. Introduction

Cadmium, a highly toxic heavy metal, tends to accumulate in the food chain. The growth of industries like mining and electroplating in recent years has led to an increase in the release of wastewater containing Cd. This ongoing discharge has resulted in the accumulation of cadmium elements in rivers and coastal waters [1]. The Pacific oysters *Crassostrea gigas* primarily inhabit marine environments including estuaries, bays, and intertidal zones along the Pacific coast. Due to their high bioaccumulation capacity, they are capable of accumulating 104 times more Cd in their soft tissues compared to the concentration found in seawater [2,3]. Consequently, Cd pollution poses certain risks to human health and other predators’ food safety while also causing economic losses for the aquaculture industry [4]. Currently, marine Cd pollution is recognized as a global issue with significant implications for marine organisms.

Most bivalves are benthic filter feeders with a strong ability to accumulate heavy metals. Due to deficiencies in their metabolic mixed-oxidase systems, they release internal pollutants more slowly than fish and crustaceans, resulting in higher levels of accumulation in their bodies, which significantly affects growth, immunity, metabolism, and other processes [5,6]. Despite the elevated levels of heavy metals in the oysters’ bodies, they do not exhibit apparent tissue deformities, suggesting that they possess specialized mechanisms for accumulating, storing, and detoxifying heavy metals. Research on the heavy metal tolerance mechanisms of bivalves has been increasing annually, mainly focusing on aspects such as toxicology, immunology, antioxidant enzyme activity, functional genes, and DNA damage [7,8,9,10].

Marine species contaminated with heavy metals tend to accumulate substantial quantities of these metals, which markedly impact their growth, immunity, metabolism, and various other physiological processes. For instance, the Mediterranean mussel *Mytilus galloprovincialis* shows significant responses to Cd exposure by increasing metallothionein levels and antioxidant enzyme activity, which are biomarkers of metal exposure [11]. In the clam *Ruditapes philippinarum*, Cd exposure increases the energy demand under cadmium stress by upregulating glycolysis and the TCA cycle pathways. [12]. Additionally, the freshwater mussel *Anodonta woodiana* exhibits DNA damage and protein denaturation from cadmium exposure but mitigates these effects by activating repair genes and antioxidant defenses, demonstrating cadmium tolerance [13]. Although different bivalves adopt various strategies to cope with cadmium pollution, they all exhibit adaptation and resistance mechanisms. However, there have been few studies on the response mechanisms of mollusks under acute Cd exposure, and none specifically on oysters.

Metabolomics is a technique used to examine the qualitative and quantitative aspects of small molecule metabolites present in an organism after undergoing perturbations, such as alterations in genetics or surroundings. It aims to identify the types of metabolites and their patterns of change and to elucidate the relationships between metabolites and physiological changes [14]. Transcriptomics refers to a methodology that studies gene transcription and transcriptional regulatory patterns at the global level within cells. It involves the investigation of gene expression at the RNA level [15].

In bivalves, heavy metals tend to accumulate primarily in their gill tissues, which exhibit significant responses to metal toxicity [16]. The gills of bivalve mollusks are primary target organs for metal accumulation due to their direct contact with heavy metals in water, often exhibiting the highest levels of pollutants [17]. Studies have shown that among the gills, hepatopancreas, and mantle of *Meretrix meretrix*, the gills contain the highest levels of Cd. Furthermore, dissolved metals are primarily absorbed through direct adsorption onto the gills and mantle, while particulate metals tend to be absorbed via the digestive organs along with food [18]. The gills also serve as a major barrier against damage and pathological factors mediated by environmental pollutants, making them the first site of toxic metal impact [19]. Therefore, we chose to analyze the gill tissues of oysters for subsequent analysis. By employing a combined approach of transcriptomics and metabolomics, we can uncover the correlations between genes and metabolites, identify key regulatory nodes of genes and metabolites, and elucidate the regulatory mechanisms of metabolic pathways within organisms. Our study aims to provide further evidence of the toxicological mechanisms occurring in oysters under acute Cd exposure.

In this study, we used the Pacific oyster *C. gigas* as a toxicological model to investigate the molecular mechanisms of molecular response and metal toxicity under acute Cd exposure, and we revealed the oxidative stress response induced by acute Cd exposure in the oyster. Through the enrichment analysis of transcriptome and metabolome data, we also studied the toxicity effects of acute Cd exposure on the oyster and the mechanisms regulating the strength of tolerance from the translational and metabolic levels. Through these analyses, our objectives were to (1) clarify the molecular mechanisms of oxidative stress and energy metabolism disorders in oysters under acute Cd exposure, and (2) explore the molecular mechanisms and potential molecular mechanisms of high Cd tolerance response in *C. gigas*. Our results have provided fundamental insights into the regulatory pathways of complex Cd responses.

## 2. Materials and Methods

### 2.1. Animal Materials and Cd Treatment

*C. gigas* oysters were sourced from the Kongtong Island Breeding Base in Yantai, Shandong Province, China, and underwent a one-week adaptation period in a seawater tank. These one-year-old *C. gigas* had an average shell length of 69.07 mm (range: 61.92–76.23 mm) and an average weight of 45.71 g (range: 39.84–54.68 g) and were not in the breeding season. During both the adaptation phase and subsequent exposure to Cd, the oysters were kept in seawater filtered to maintain a temperature of 24 ± 2 °C. To minimize the introduction of metals through the food chain, they received a diet of freshly harvested microalgae (comprising both *Diatom* and *Chlorella vulgaris*) every alternate day. The oysters were allocated across six 60 L tanks, each containing seawater that had been filtered (three biological replicates for both the experimental and control groups), with 10 oysters per tank. 

A dilution of Cd was created by dissolving CdCl_2_ in deionized water, achieving a concentration of 1 mg/L. The Cd concentrations were determined based on previous studies and our preliminary research. After a pre-experiment, the 96 h LC50 (semi-lethal concentration) was determined to be 65.75 mg/L. The stock solution was added to three tanks in the experimental group, with the Cd concentration in acute exposure set at one-twentieth of the LC50, resulting in a final seawater Cd concentration of 3.4 mg/L [20]. Furthermore, concentrations higher than environmental standards were employed to provoke a significant stress response in the organism, thereby guaranteeing the validity of the results. In a study, the concentration of cadmium in the treatment group for greenfin horse-faced filefish (*Thamnaconus septentrionalis*) was set at 50 mg/L [21]. Therefore, in research on the response of oysters to cadmium stress, a cadmium concentration of 3.4 mg/L in seawater is considered a reasonable level. The oysters were exposed to Cd for 96 h. Throughout the experiment, the seawater was changed daily, and the survival status of the oysters was monitored. Deceased individuals were promptly removed and recorded. For sample collection, gill tissues were sampled from three oysters in each of the six tanks (acute Cd exposure and control groups). The samples were promptly cryogenically preserved in liquid nitrogen and stored at a temperature of −80 °C. These samples were reserved for subsequent analyses, including transcriptomics, metabolomics, and the determination of physiological and biochemical indicators.

### 2.2. Cd Concentration and Biochemical Parameter Analysis

Gill samples weighing 0.5 g were first homogenized, lyophilized, and then completely digested with a mixture of HCl (20%) and HNO_3_ (80%). These samples were sealed and heated in an oven at 150 °C for a duration of 10 h. Upon cooling, the concentration of Cd in the oyster gill tissues was assessed using inductively coupled plasma mass spectrometry (ICP-MS) (Thermo Scientific iCAP Q ICP-MS, manufactured in Waltham, MA, USA). Additionally, 0.1 g of gill tissue from both groups (the Cd exposure group and the control group) were homogenized in a chilled phosphate buffer solution. The mixture was centrifuged at a speed of 3000× *g* for 15 min, resulting in the collection of the supernatant. Commercial assay kits were employed to quantify enzyme activity levels, including superoxide dismutase (SOD), catalase (CAT), malondialdehyde (MDA), and glutathione peroxidase (GSH-PX), within the collected supernatant [22]. The overall quantification of soluble proteins was conducted using the Bradford technique, with bovine serum albumin (BSA) serving as the standard reference, and measurements were recorded at a wavelength of 595 nm. Enzyme assays were conducted in triplicate to ensure accuracy and reproducibility.

### 2.3. Metabolite Extraction, Detection, and Analysis

Both control individuals and those exposed to acute Cd (9 samples/group) had their gill tissues cryopreserved in liquid nitrogen before being air-dried and pulverized into fine powder. This powder was then reconstituted in an 80% methanol solution and diluted with LC-MS-grade water to reach a final concentration of 53% methanol. After centrifugation (1500 g, 4 °C, 20 min), the resulting supernatant was analyzed using the LC-MS system. Metabolite annotation was carried out leveraging databases such as KEGG and LIPIDMaps. To analyze the data, we utilized MetaX 1.4.2 Software, which is specifically designed for interpreting metabolomics data and includes principal component analysis (PCA) and partial least squares discriminant analysis (PLS-DA). Statistical significance was determined using univariate analysis based on *p*-values. Metabolites showing variable importance in projection (VIP) > 1, *p*-value < 0.05, and fold change (FC) ≥ 2 or FC ≤ 0.5 were classified as DEMs. The roles and associated metabolic pathways of these metabolites were explored using the KEGG database. Pathway enrichment analysis of differential metabolites was conducted, deeming a pathway significantly enriched when the ratio x/n exceeded y/N, and the pathway’s *p*-value was <0.05.

### 2.4. Sampling, RNA Extraction, Library Construction, and RNA-Seq Analysis

RNA was extracted from the gill tissues of nine oysters per group, with every three oysters pooled as one sample, for RNA sequencing of both the acute Cd exposure and control groups. Subsequent analyses utilized only high-quality, clean data. A reference genome index was built, and the clean, paired-end reads were mapped to this reference using Hisat2 v2.0.5 [23]. The FPKM metric was used to quantify gene expression levels. To control the false discovery rate and adjust *p*-values, we employed the Benjamini and Hochberg method. In our study, a *p*-value ≤ 0.05 and |log_2_ (fold change)| ≥ 1 were used as the thresholds to define DEGs. Enrichment analyses for the DEGs were executed using DAVID v6.8, leveraging the reference genome as background. The DEGs served as the validation set for GO and KEGG analyses, enabling the assessment of DEG distribution in various functions and pathways between the acute Cd-exposed and control groups.

### 2.5. Expression Validation Using Quantitative Real-Time PCR

To validate the RNA-Seq findings, qRT-PCR was conducted on 12 selected DEGs (Figure S1). Primers for these DEGs were designed using Primer Premier 6.0, and the 2^−ΔΔCt^ technique was employed to quantify their relative expression levels [24].

### 2.6. Integrative Analysis of Metabolome and Transcriptome

DEGs (*p*-value < 0.05 and |log2FC| > 1) and DEMs (VIP > 1 and *p* < 0.05) were used for the integrative analysis of the acute Cd exposure and control groups. The Spearman method analyzed the correlation coefficients for integrating the metabolome and transcriptome data. A heat plot visually represented the relationships between genes and metabolites.

### 2.7. Statistical Analysis

The comparison of Cd concentration and biochemical parameters between the acute Cd exposure and control groups was performed using a *t*-test, with a significance threshold set at *p* < 0.05. This analysis was conducted using SPSS 19.0 software.

## 3. Results

### 3.1. Cd Concentration and Biochemical Parameter Analysis

**Figure 1:**
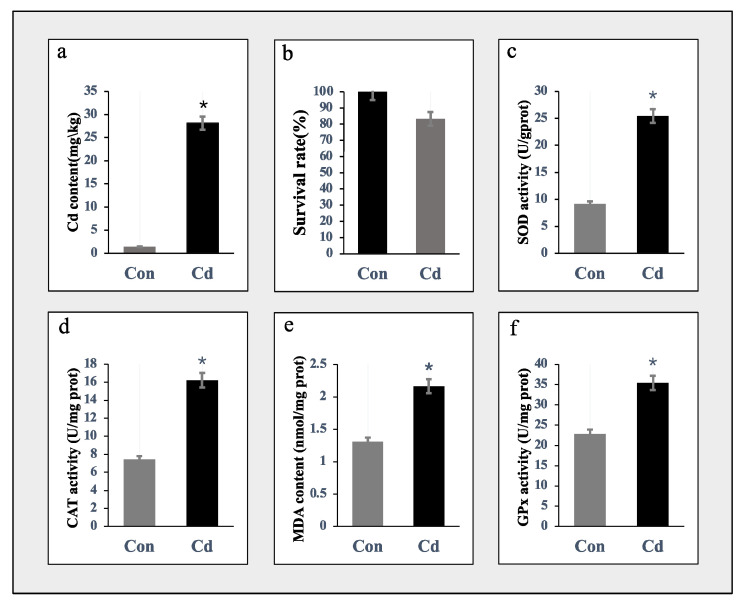
Biochemical alterations of the acute Cd exposure *C. gigas*. (**a**) Increased intracellular Cd level in Cd-exposed oyster individuals. The bar chart depicts mean levels and standard deviation (SD) values, with *n* = 3. Significance differences were determined by *t*-test (*p* < 0.05), denoted by asterisks (*). (**b**) The survival rate of oysters after 96 h of acute Cd exposure. (**c**–**f**) After Cd exposure, oxidative stress markers such as SOD (**c**), CAT (**d**), MDA (**e**), and GPx (**f**) were measured. Data were presented as the mean ± SD (*n* = 3). Distinguishing letters were assigned to indicate significant differences (*p* < 0.05). Star plots depict the impact of Cd exposure on gill samples.

Figure 1a shows that the concentration of Cd in the gill tissues of oysters was 28-fold higher in the acute exposure group compared to the control group. Figure 1b demonstrates that the survival rate of oysters after 96 h of acute Cd exposure was 83.3%. Our preliminary experiment revealed that adult oysters exhibit a remarkable tolerance to acute cadmium stress, surpassing that of other species. Among these, the activities of SOD, CAT, and GPx in the gill tissues of the Cd-treated group significantly increased (Figure 1c,d,f), along with a significant elevation in MDA content (Figure 1e). The results indicate a significant increase in these enzyme activity indices, preliminarily suggesting that acute Cd stress leads to an augmentation of oxidative stress and an enhanced molecular response within oyster gill cells.

### 3.2. Transcriptomic Changes Induced by Acute Cd Exposure

**Figure 2:**
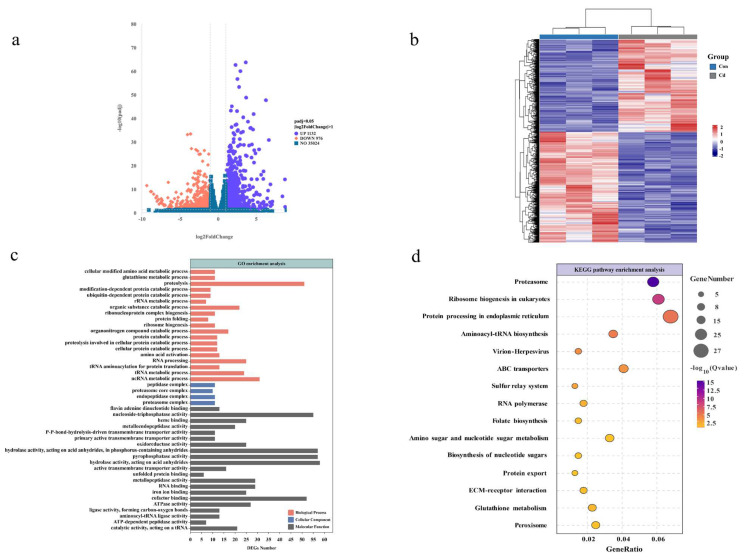
Analysis of oyster transcriptome following a 96-hour exposure to Cd. (**a**) A volcano plot of DEGs is graphically represented with upregulated genes in red and downregulated genes in blue. (**b**) Hierarchical clustering based on the DEGs, where red signifies upregulation and blue signifies downregulation. (**c**) Enrichment of DEGs in GO terms categorized into cellular components, biological processes, and molecular functions. (**d**) KEGG pathway enrichment analysis of DEGs, with colors indicating *p*-value significance and bubble size reflecting the count of enriched genes.

The volcano plot visually represents the distribution of DEGs between the groups treated with Cd and those under control conditions. Compared to the control group, the exposed oysters exhibited 1132 genes upregulated and 976 genes downregulated internally (Figure 2a). A visual representation of the hierarchical clustering analysis conducted on all the DEGs revealed distinct segregation in response to Cd exposure (Figure 2b). To investigate the relationship between the DEGs and cell function, we performed enrichment analysis of GO and KEGG pathways. As shown in Figure 2c, significantly enriched GO terms include proteolysis, glutathione metabolic process; protein catabolic process; amino acid activation; organic substance catabolic process; tRNA metabolic process (ontology: biological process); proteasome complex; endopeptidase complex; proteasome core complex; peptidase complex (ontology: cellular component); catalytic activity, acting on a tRNA; ATP-dependent peptidase activity; cofactor binding; oxidoreductase activity; and metalloendopeptidase activity (ontology: molecular function).

The enrichment of level 2 KEGG signaling pathways such as proteasome, protein processing in the endoplasmic reticulum, ABC transporters, sulfur relay system, amino sugar and nucleotide sugar metabolism, protein export, ECM–receptor interaction, glutathione metabolism, and the peroxisome pathway (Figure 2d). However, this does not definitively establish that these physiological processes occurred within the organism. Therefore, we proceed to further examine the metabolome to ascertain whether metabolic pathways or metabolites relevant to the transcriptome results can be identified, thereby confirming the occurrence of physiological activities induced by acute Cd exposure within oyster gill cells.

### 3.3. Metabolomic Changes Induced by Acute Cd Exposure

**Figure 3:**
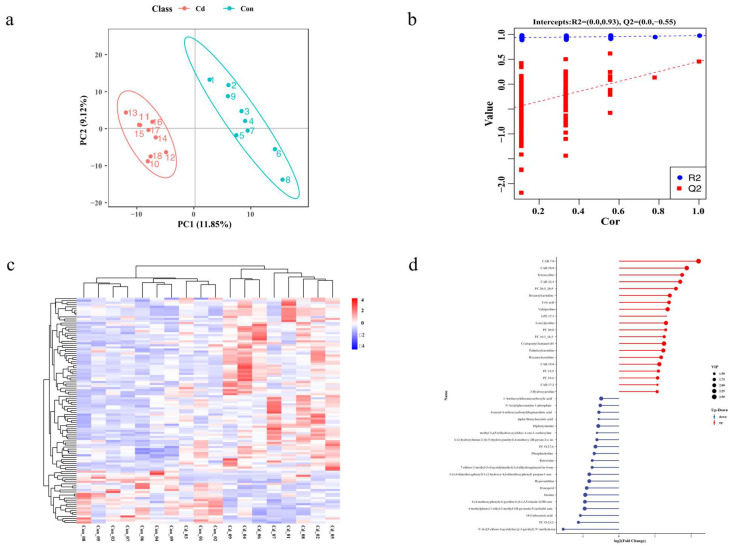
Analysis of oyster metabolomes following a 96-hour exposure to Cd. (**a**) OPLS-DA score plot of the metabolomic data. (**b**) PLS-DA sorting test plot of the metabolomic data. (**c**) Hierarchical clustering was performed using 111 DEMs, with red indicating upregulation and blue representing downregulation, respectively. (**d**) Sample comparisons for the matchstick diagram. The top 20 metabolites of up and down are displayed in the matchstick diagram. The *x*-axis of the matchstick diagram represents log_2_ (Fold Change) values, the *y*-axis represents metabolites, and the size of the points corresponds to VIP values. The metabolites that are upregulated and downregulated are represented by the red and blue points, respectively.

**Figure 4:**
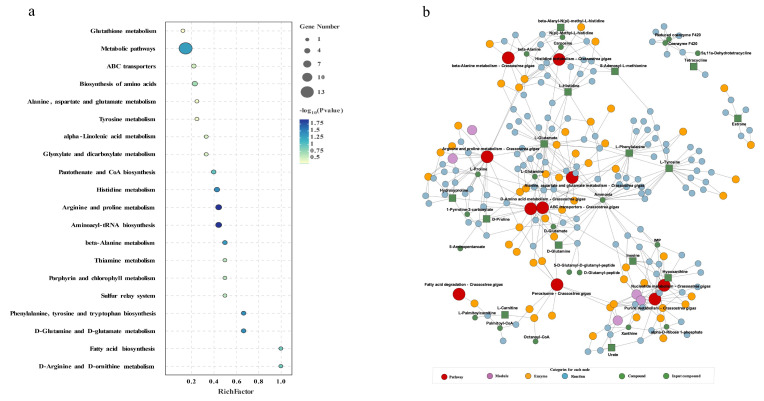
Pathway analysis of the DEMs. (**a**) Top 20 KEGG pathway results. *p*-values are represented by colors, while pathway impact is indicated by the size of the bubbles. (**b**) KEGG regulatory network diagram. Red circles represent individual metabolic pathways, yellow circles depict enzyme information related to specific substances, green circles indicate background substances for a metabolic pathway, purple circles represent information on molecular modules of a certain substance category, blue circles represent chemical interactions involving a specific substance, and green squares denote differentially expressed substances identified in this comparison.

To examine metabolic alterations following acute Cd exposure, intracellular metabolites were scrutinized using an LC-MS system. Distinct metabolomic profiles emerged in the OPLS-DA model after Cd exposure (Figure 3a,b). A total of 111 differentially expressed metabolites (DEMs) were identified, characterized by VIP values > 1.0 and *p*-values < 0.05. Among these, the Cd-exposed group exhibited elevated levels in 85 metabolites, whereas decreased levels were observed in 26 metabolites (Figure 3d). The unsupervised hierarchical clustering analysis of the 110 differentially expressed metabolites further emphasized the distinct expression patterns between the two groups. (Figure 3c). To delve deeper into the disrupted metabolic pathways, KEGG pathway analysis was conducted using MetaboAnalyst 5.0. The result showed the top 20 significantly enriched KEGG pathways (Figure 4a). Of these, we identified metabolic pathways related to Cd exposure, including glutathione metabolism, arginine and proline metabolism, fatty acid biosynthesis, sulfur relay system, and ABC transporters, which were significantly altered (Figure 4b).

### 3.4. Integrated Analysis of Metabolomic and Transcriptomic Data

**Figure 5:**
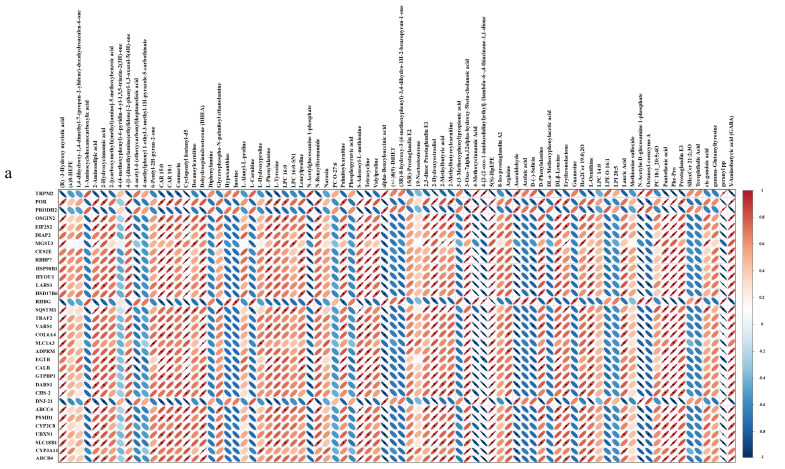
Correlation heatmap analysis of DEGs and DEMs. The DEMs are depicted on the *y*-axis, while the *x*-axis displays the DEGs. A correlation coefficient less than 0 is indicative of a negative correlation, and a coefficient greater than 0 indicates a positive correlation. Negative correlations are symbolized by the color blue, while positive correlations are represented by the color red.

**Figure 6:**
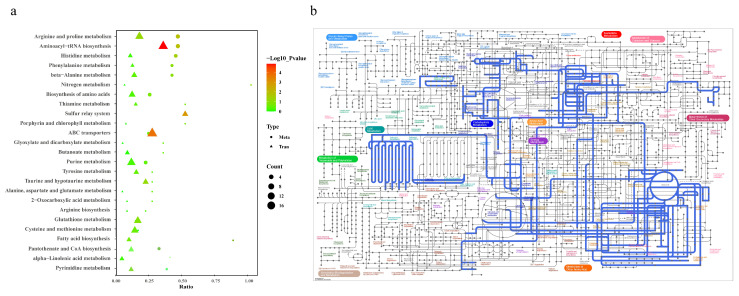
Pathway enrichment analysis of DEGs and DEMs. (**a**) Bubble plot of KEGG enrichment for DEGs and DEMs. The x-axis represents the ratio of the number of enriched differential metabolites or genes annotated to metabolites or genes in the pathway to the total number in that pathway (ratio). The *y*-axis represents KEGG pathways jointly enriched in the metabolome and transcriptome. The count indicates the number of enriched metabolites or genes in the pathway. The colors represent *p*-values, with brighter colors indicating smaller *p*-values and more significant pathway enrichment. (**b**) iPath pathway map of shared enriched pathways. Colored boxes represent enriched pathways, nodes depict various biochemical molecules, lines represent biochemical reactions, and blue lines within the pathways indicate pathways jointly enriched with DEGs and DEMs.

To conduct a comprehensive analysis of the impact of Cd on biochemical pathways using systems biology methods, we performed a joint-pathway analysis in MetaboAnalyst by integrating the DEMs and DEGs at the pathway level. Figure 5 shows the results of the correlation analysis of the top 82 DEMs and the top 33 DEGs. The data shows that a total of 25 pathways were enriched, including arginine and proline metabolism, aminoacyl-tRNA biosynthesis, histidine metabolism, phenylalanine metabolism, beta-alanine metabolism, nitrogen metabolism, the biosynthesis of amino acids, thiamine metabolism, the sulfur relay system, porphyrin and chlorophyll metabolism, ABC transporters, glyoxylate and dicarboxylate metabolism, butanoate metabolism, purine metabolism, tyrosine metabolism, taurine and hypotaurine metabolism, alanine, aspartate, and glutamate metabolism, 2-oxocarboxylic acid metabolism, arginine biosynthesis, glutathione metabolism, cysteine and methionine metabolism, fatty acid biosynthesis, pantothenate and CoA biosynthesis, alpha-linolenic acid metabolism, and pyrimidine metabolism (Figure 6a). 

**Figure 7:**
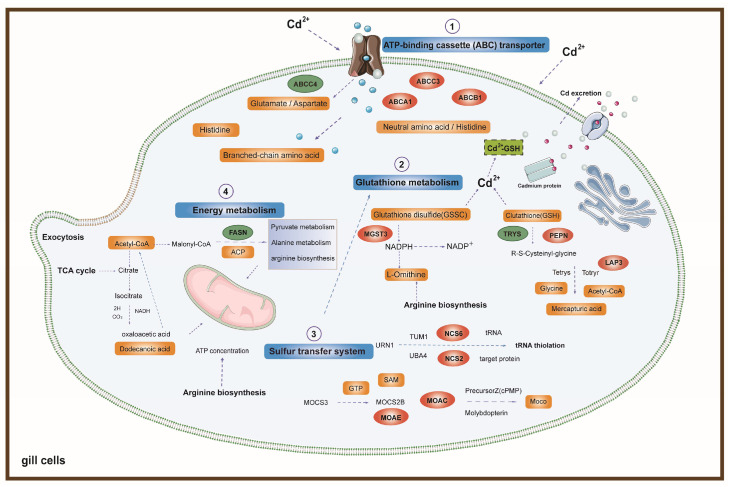
Main biological pathway responses to acute Cd exposure in oysters. The blue boxes represent four core Cd exposure response pathways. The orange boxes represent DEMs associated with the main pathways. The green boxes represent downregulated DEGs related to the main pathways. The red elliptical frames represent upregulated DEGs associated with the main pathways.

The construction and visualization of the gene-metabolism network were carried out utilizing Metscape. The differential genes and metabolites were mapped to the KEGG database for identification of common pathways, leading to the discovery of key biochemical and signal transduction pathways associated with these molecules. The results indicated energy metabolism, amino acid metabolism, carbohydrate metabolism, metabolism of terpenoids and polyketides, glutathione metabolism, glyoxylic acid and dicarboxylic acid metabolism, the sulfur relay system, and ABC transporters were significantly enriched (Figure 6b). We further categorized these physiological processes in conjunction with the iPath diagram to elucidate the primary impact of Cd exposure. The bold blue lines in the iPath pathway map represent commonly enriched pathways identified by both omics analyses. Among them, the ABC transporter, glutathione metabolism, sulfur relay system, and energy metabolism were found to be significant. We selected these four pathways that exhibited the strongest association with Cd exposure for further investigation. Furthermore, we selected 12 core genes for qPCR validation. The results demonstrated that the changes in gene expression levels were consistent with those observed in the transcriptome analysis, ensuring the reliability of the findings (Figure S1). By integrating DEMs and DEGs from these pathways, we attempted to identify the core genes and metabolites responsive to acute Cd exposure. These four pathways were significantly enriched in both the transcriptomic and metabolomic results. Based on our findings, we constructed a comprehensive toxicological mechanism map encompassing differential genes and metabolites involved in these pathways as well as their intricate interactions (Figure 7).

## 4. Discussion

### 4.1. The Toxicological Response Mechanisms of Oysters under Cd Exposure

Transcriptomics is a pivotal approach for investigating cellular functions and examining gene expression at the RNA level. Metabolomics unveils mechanisms underlying biological activities by elucidating patterns of changes in small-molecule metabolites. Through correlated analysis of transcriptomics and metabolomics, critical metabolic pathways and regulatory genes can be more accurately identified, thereby providing deeper insights into the response mechanisms within oysters under acute Cd stress [25]. The oyster gill tissue exhibits a specific response to Cd exposure, acting as a primary barrier against damage and pathological factors [26]. When gill cells face acute cadmium exposure, they undergo a series of physiological and biochemical changes. In our transcriptomic and metabolomic KEGG results, we observed significant changes in the following pathways. The enhancement of the ABC transporter pathway in the gill tissues indicates its critical role in cadmium efflux. Kim et al. demonstrated that ABC transporters are significantly activated under cadmium exposure, accelerating cadmium efflux and reducing intracellular toxic accumulation [27]. Furthermore, we found significant changes in the glutathione metabolism within gill tissues. Under cadmium stress, gill tissues showed a marked increase in GSH levels, indicating a high capacity for synthesizing and utilizing GSH to counteract cadmium-induced oxidative stress, thus enhancing detoxification and protective mechanisms. This differential response highlights the unique role of gills in managing oxidative damage. Similarly, Espinoza et al. emphasized the importance of GSH in the detoxification of heavy metal Cd in the gill tissues of coho salmon *Oncorhynchus kisutch* [28]. Additionally, the energy metabolism pathways in gill tissues also exhibited significant alterations. Our study detected notable changes in metabolic pathways related to ATP production and utilization, indicating that cadmium exposure disrupts the energy homeostasis in gill cells. This forces the cells to alter gene expression to support detoxification processes and damage repair mechanisms. Zhang et al.’s research supports this view, showing that gill cells in greenfin horse-faced filefish *Thamnaconus septentrionalis* adjust glycolysis, protein degradation, and fatty acid metabolism to meet the nutritional and energy demands under cadmium stress [21,29].

Our study uses an integrated approach combining transcriptomic and metabolomic analyses to gain a deeper and more comprehensive understanding of the acute toxicological mechanisms in Pacific oysters. It reveals the unique response mechanisms of oyster gill tissues to Cd exposure, including significant changes in ABC transporter proteins, glutathione metabolism, energy metabolism, and oxidative damage pathways. These findings not only confirm previous research results but also provide new insights into the molecular mechanisms underlying the acute stress responses of aquatic invertebrates to heavy metal Cd exposure. While it is well-known that oysters exhibit extreme tolerance to cadmium, our focus is to observe the significant molecular changes during acute cadmium stress. This approach aims to identify the most critical physiological changes and uncover the survival strategies oysters employ to cope with high cadmium concentrations. Understanding these immediate molecular responses is important for determining key biomarkers and physiological pathways involved in their remarkable tolerance.

#### 4.1.1. The Role of ABC Transport Proteins in Cd Tolerance in Oysters

During acute Cd stress, oyster gill cells exhibit an immediate response in both their cell membranes and organelles [30]. Among these, ATP-binding cassette (ABC) transport proteins constitute a class of membrane proteins that play crucial roles in transport and efflux functions on the cell membrane [31] (Figure 7). The ABC transporters have a crucial function in cellular detoxification as they actively remove harmful substances and their byproducts from the cell through ATP-dependent binding and hydrolysis mechanisms [32]. The ABC transport mechanism has been identified and characterized in various aquatic multicellular eukaryotes, including bivalve mussel *Mytilus edulis* [33,34], teleost fish [35,36,37], and crustaceans [38,39].

Previous studies have indicated that the activation of ABC transport proteins in bacteria, plants, and animals is linked to protective mechanisms against the toxic metal cadmium. These proteins have shown their capability to alleviate the detrimental impacts of Cd exposure on individual development and viability [40,41,42]. When the organism is subjected to cadmium stress, ABC transport proteins act as cadmium efflux pumps, enhancing Cd tolerance by reducing the intracellular Cd content [43]. The activity and gene expression of ABC transport proteins have been identified in a range of aquatic organisms, serving to respond to changes in environmentally exogenous substances [44,45]. Studies employing gene knockout have demonstrated that ABC transporters play a pivotal role in *Arabidopsis thaliana*, yeast *Saccharomyces cerevisiae*, and marine ciliates *Paramecium marinus*, enhancing the organisms’ Cd efflux rate and tolerance [46,47,48]. ABC transport proteins are considered key components of the multidrug resistance (MDR) system, capable of blocking the uptake of compounds by cells and controlling the efflux of metabolites. Abcb1 is a gene that synthesizes MDR-related ABC transport proteins. In aquatic invertebrates, exposure to 5 μg/L Cd upregulates the expression of ABCB1 in the gill tissue of the clam *Ruditapes philippinarum*, significantly activating this transporter and expediting the efflux of Cd^2+^ [49,50]. 

In addition to the detoxification role of ABC transport proteins, acute Cd exposure is also associated with a significant molecular response in mollusks. For instance, the molecular response in oyster gill cells involves the upregulation of genes related to oxidative stress pathways. SOD is an antioxidative enzyme utilized for scavenging superoxide radicals, representing highly deleterious oxidative species. Elevated SOD activity signifies enhanced capacity within the oyster organism to combat oxidative stress [51]. CAT primarily functions to degrade hydrogen peroxide, a toxic oxidative substance. Elevated CAT activity indicates an enhanced efficiency of oyster gill cells in clearing hydrogen peroxide [52]. MDA serves as a lipid peroxidation byproduct, typically utilized as a marker for oxidative stress. Glutathione peroxidase (Gpx), with its principal role in degrading peroxides, including lipid peroxides, signifies an elevated defense against oxidative stress within oyster gill cells under acute Cd exposure when its activity increases [53]. The elevation in MDA levels signifies an increased degree of membrane lipid oxidation in oyster gill cells under Cd treatment, indicating a certain level of oxidative damage to the cell membrane. Enzyme activities significantly increase, indicating an enhanced antioxidant defense mechanism.

In this study, we observed a significant upregulation of genes such as *ABCA1*, *ABCB1*, and *ABCC3*, along with a notable downregulation of the *ABCC4* gene under acute Cd exposure. Associated differential metabolites included glutamate, aspartate, branched-chain amino acids, neutral amino acids, and histidine. Our findings indicate that under acute Cd stress, genes associated with ABC transport proteins are expressed in oyster gill cells, leading to the generation of related metabolites. This suggests a crucial role of ABC transporters in alleviating Cd toxicity and maintaining the balance of cadmium concentrations within oyster gill cells. Moreover, the high expression of these ABC transporter-related genes and associated metabolites may contribute to the oyster’s high accumulation and tolerance to Cd, reflecting its adaptive response to Cd exposure.

#### 4.1.2. Glutathione Metabolism Plays a Detoxifying Role Against Cd

Upon the ingress of cadmium ions into the cellular environment, a series of physiological reactions is triggered. Oysters exhibit an exceptional tolerance and accumulation capacity for Cd, which indicates that Cd does not predominantly exist in a free state within the cells. It is inferred that oysters possess certain proteins capable of binding with Cd to form complexes, enabling Cd to remain in a stable state within the cell. This mechanism significantly mitigates the cytotoxic effects of Cd. Our findings reveal that the secondary pathway, glutathione metabolism, possesses this capability (Figure 7). It executes vital antioxidative and detoxification duties within the cellular environment, especially in combating oxidative stress induced by heavy metals like Cd [54]. Glutathione (GSH) represents a critically important internal antioxidant, with a plethora of essential physiological roles such as free radical elimination and detoxification, contributing significantly to the organism’s biochemical defense mechanism [55]. Previous research has demonstrated the protective role of glutathione (GSH) in isolated fish gill cells against the cytotoxicity of metal ions. Cd can significantly increase the depletion of GSH in cells, with GSH acting as a direct chelator for intracellular metal ions. Metal ions, due to their strong affinity for sulfur, can bind to the thiol groups within GSH molecules. A portion of this remains inside the cell as stable metal chelate proteins, while another is expelled through transport proteins on the cell membrane, thus facilitating detoxification and reducing the loss inflicted by heavy metals on cells [56,57]. Furthermore, glutathione functions as an essential cofactor for glutathione peroxidase, facilitating the breakdown of hydrogen peroxide and lipid peroxides generated during redox processes [58]. In this study, the increase in glutathione transferase levels may be attributed to the activation of the glutathione metabolic pathway. The most common oxidized form of glutathione in cells is glutathione disulfide (GSSG). Previous research has indicated that the GSH/GSSG redox buffer participates in many vital cellular functions, including cell differentiation, proliferation, apoptosis, ferroptosis, signal transduction, cytokine production, and molecular responses [59]. The initial and pivotal step in glutathione biosynthesis involves the coupling of glutamate and cysteine, facilitated by glutamate–cysteine ligase. Subsequently, the produced γ-glutamylcysteine combines with glycine, culminating in the formation of GSH by the enzyme GSH synthetase [60]. In this research, the expression of genes related to the glutathione metabolic pathway, such as *PEPN*, *LAP3*, and *MGST3*, was significantly increased, whereas the expression of *TRYS* was significantly decreased. Differential metabolites include Glutathione disulfide (GSSG), Glutathione, R-S-Cysteinyl-glycine, Mercapturic acid, and L-Ornithine. These genes may influence the pathways associated with glutathione metabolism, thus enhancing the survival rate and tolerance of oysters under acute Cd stress and leading to the generation of related metabolites.

The sulfur relay system constitutes the third pathway, functioning in the thiolation of sulfur within tRNA and the pathway for cysteine production. A tight interplay exists between this system and glutathione metabolism, collaboratively ensuring the equilibrium of sulfur metabolism and antioxidative protection within cells. The sources of sulfur for the sulfur relay system comprise an array of enzymes and molecules connected to sulfur, like cysteine, methionine, and cystathionine, among others [61]. These molecules are precursor substances for the synthesis of glutathione. Among them, cysteine is a critical component in the synthesis of glutathione because it contains sulfur, serving as the sulfur source for glutathione molecules. In European seabass (*Dicentrarchus labrax*), alterations in the gut microbiota impact immune status and digestive performance. The organism can adapt to these changes through the regulation of the sulfur relay system [62]. Glutathione is synthesized from cysteine, cystathionine, and methionine, with both cysteine and cystathionine being directly related to the sulfur relay system. These precursor substances undergo a series of enzymatic reactions to ultimately produce glutathione. The sulfur relay system, by providing a source of sulfur, maintains the sulfur metabolic balance within oysters, sustains glutathione levels, and enhances antioxidative defense [63,64]. In this study, genes related to the sulfur relay system, such as *NCS2*, *NCS8*, *MOAE*, and *MOAC*, were significantly upregulated. Differential metabolites include GTP, SAM, and MOCO. This indicates that in oysters under acute Cd exposure, pathways related to the sulfur relay system are activated and work in conjunction with the glutathione metabolic pathway to maintain intracellular antioxidative capacity, playing a critical role in cellular homeostasis.

Our results underscore the critical role of the glutathione metabolic pathway in this process, as it exerts important antioxidative and detoxifying effects within the cellular environment. It serves as a significant internal antioxidant, playing a central role in combating oxidative stress induced by heavy metals such as cadmium. The upregulation of genes associated with glutathione metabolism and the activation of the sulfur relay system enhance the survival and tolerance of oysters under acute cadmium exposure, thereby strengthening the molecular response of oysters to cadmium-induced oxidative damage. This interconnected network of pathways emphasizes the complex biological strategies adopted by oysters to counter cadmium stress, reflecting their ability to enhance immunity and maintain cellular homeostasis in the face of heavy metal toxicity. Understanding these interrelated mechanisms provides fundamental information for studying defense mechanisms and cadmium tolerance in mollusks.

#### 4.1.3. Acute Cadmium Exposure Affects Gill Energy Metabolism

The maintenance of physiological functions and the regulation of stress heavily rely on energy metabolism. Typically, organisms are adequately supplied with energy to meet their requirements, while any excess is stored [65]. However, the energy balance of marine organisms can be disturbed by heavy metal stress. As a result, organisms may need to expend additional energy in order to activate stress responses, facilitate detoxification processes, and engage in damage repair mechanisms [66]. In energy metabolism, glycolysis is a crucial pathway for glucose metabolism. Glycolysis is the process of breaking down a glucose molecule into two pyruvate molecules, resulting in a net production of two ATP molecules and two NADH molecules. In the presence of oxygen, pyruvate enters the mitochondria, where it undergoes further metabolism via the TCA cycle and oxidative phosphorylation, leading to a significant generation of ATP [67]. We discovered that in the Cd-treated groups, the levels of metabolites involved in glycolysis significantly increased, such as dodecanoic acid, acetyl-CoA, and ACP. In addition, the downregulation of the FASN gene caught our attention, indicating that the energy metabolism process was promoted under Cd exposure (Figure 7). Previous research has found that with the increase in Cd concentration in mice, the glycolysis pathway was significantly enhanced [68].

Furthermore, Cd can trigger oxidative stress reactions within cells, resulting in an overproduction of oxygen free radicals. These radicals can harm biomacromolecules within cells, such as proteins, lipids, and DNA, consequently disrupting normal cellular metabolism [69,70,71,72]. In our results, Cd exposure induced changes in fatty acid metabolism, alanine, and arginine metabolism. Alanine is an energy substrate primarily involved in the tricarboxylic acid cycle, while arginine primarily maintains stable ADP concentrations and can act as an energy buffer in tissues requiring high energy. In the study of rainbow trout Oncorhynchus mykiss, it was observed that the production of fatty acids and arginine under Cd exposure affected cellular homeostasis and metabolism [73,74]. Our study also indicates that the inhibitory effect of Cd exposure may lead to disruptions in the energy metabolism pathways of oyster organisms and oxidative stress damage.

The tricarboxylic acid cycle is a sequence of biochemical reactions employed by aerobic organisms to produce energy via the oxidation of acetyl-CoA obtained from carbohydrates, fatty acids, and proteins [75]. Our results reveal that metabolomic analysis showed an accumulation of free fatty acids and some small peptide molecules, such as dodecanoic acid, acetyl-CoA, and ACP. It has been reported that Cd exposure alters the abundance of metabolites related to energy metabolism (ATP, ACP, phosphatidylcholine, lactate, and succinate) in the liver of the halibut *Paralichthys olivaceus* [76]. In our results, we also detected relevant metabolites among which the increased levels of ACP may result in cellular damage. ACP is a lysosomal enzyme with activity in multiple biological processes, including cathepsin, pathological necrosis, autophagy, and phagocytosis. Metals can accumulate in lysosomes, leading to increased toxicity and alterations in lysosomal function or structure, consequently causing cellular damage [77,78]. In the transcriptome results, we found that the *FASN* gene was significantly downregulated. Previous studies have shown that the downregulation of *FASN* can alleviate energy metabolism disorders induced by potassium dichromate in LMH cells. This indicates that the TCA cycle pathway is activated and enhanced under Cd stress, leading to energy disruption and structural–functional changes in oyster gill cells.

In summary, Cd exposure significantly activates the glycolytic pathway, increasing the levels of metabolites involved in glycolysis. Cd stress triggers oxidative stress reactions within cells, leading to the generation of oxygen radicals that damage cellular biomolecules, resulting in alterations in fatty acid and amino acid metabolism, ultimately causing disruptions in energy metabolism and cellular dysfunction. These findings reveal the potential impact of heavy metal pollution on the energy balance and physiological functions of marine organisms.

## 5. Conclusions

This study, through a comprehensive analysis of the metabolomic and transcriptomic responses of Pacific oysters *C. gigas* to acute Cd exposure, delves into the molecular mechanisms of oysters’ response to heavy metal Cd pollution, especially in marine environments. Our results indicate that Cd exposure significantly increases Cd concentration in oyster gill tissues, eliciting strong physiological and metabolic reactions, including enhanced oxidative stress response and disrupted energy metabolism, signaling the oyster’s biological response to acute Cd stress. Furthermore, oysters can enhance their tolerance and detoxification capabilities under acute Cd exposure by activating key pathways such as ABC transporters, regulating glutathione metabolism, participating in the sulfur relay system, and adjusting energy metabolism, thereby bolstering their molecular response to Cd-induced cellular damage. Our study not only offers new perspectives on the acute biological responses of marine organisms like bivalves to heavy metals but also provides important scientific evidence for the study of the biological effects and mechanisms of heavy metal pollution in marine ecosystems.

## Data Availability

The raw data supporting the conclusions of this article will be made available by the authors upon request.

## Supplementary Materials

The following supporting information can be downloaded at: https://www.mdpi.com/article/10.3390/ani14162318/s1. Figure S1. Comparison of qPCR and RNA-Seq results of 12 hub genes. Expression levels of key genes were standardized using the expression levels of β-actin genes. The *x*-axis represents the Cd-exposed group and the control group, while the *y*-axis represents the fold change in gene expression comparing the Cd-exposed group to the control group. The letters indicate the level of significant difference between groups (*p* < 0.05).

## References

[1] Kubier A., Pichler T. (2019). Cadmium in Groundwater—A Synopsis Based on a Large Hydrogeochemical Data Set. Sci. Total Environ..

[2] Burioli E.A.V., Squadrone S., Stella C., Foglini C., Abete M.C., Prearo M. (2017). Trace Element Occurrence in the Pacific Oyster *Crassostrea gigas* from Coastal Marine Ecosystems in Italy. Chemosphere.

[3] Meng J., Wang W., Li L., Yin Q., Zhang G. (2017). Cadmium effects on DNA and protein metabolism in oyster (*Crassostrea gigas*) revealed by proteomic analyses. Sci. Rep..

[4] Munksgaard N.C., Burchert S., Kaestli M., Nowland S.J., O’Connor W., Gibb K.S. (2017). Cadmium uptake and zinc-cadmium antagonism in Australian tropical rock oysters: Potential solutions for oyster aquaculture enterprises. Mar. Pollut. Bull..

[5] Barchiesi F., Branciari R., Latini M., Roila R., Lediani G., Filippini G., Scortichini G., Piersanti A., Rocchegiani E., Ranucci D. (2020). Heavy Metals Contamination in Shellfish: Benefit-Risk Evaluation in Central Italy. Foods.

[6] Mohiuddin M., Hossain M.B., Ali M.M., Kamal Hossain M., Habib A., Semme S.A., Rakib M.R.J., Rahman M.A., Yu J., Al-Sadoon M.K. (2022). Human Health Risk Assessment for Exposure to Heavy Metals in Finfish and Shellfish from a Tropical Estuary. J. King Saud Univ. Sci..

[7] Khan M.I., Zahoor M., Khan A., Gulfam N., Khisroon M. (2018). Bioaccumulation of Heavy Metals and their Genotoxic Effect on Freshwater Mussel. Bull. Environ. Contam. Toxicol..

[8] Pasinszki T., Prasad S.S., Krebsz M. (2023). Quantitative determination of heavy metal contaminants in edible soft tissue of clams, mussels, and oysters. Environ. Monit. Assess..

[9] Sarker S., Vashistha D., Sarker M.S., Sarkar A. (2018). DNA damage in marine rock oyster (*Saccostrea cucullata*) exposed to environmentally available PAHs and heavy metals along the Arabian Sea coast. Ecotoxicol. Environ. Saf..

[10] Shakouri A., Gheytasi H. (2018). Bioaccumulation of heavy metals in oyster (*Saccostrea cucullata*) from Chabahar bay coast in Oman Sea: Regional, seasonal and size-dependent variations. Mar. Pollut. Bull..

[11] Nardi A., Benedetti M., D’errico G., Fattorini D., Regoli F. (2018). Effects of ocean warming and acidification on accumulation and cellular responsiveness to cadmium in mussels *Mytilus galloprovincialis*: Importance of the seasonal status. Aquat. Toxicol..

[12] Zhang X., Li F., Ji C., Wu H. (2023). Toxicological mechanism of cadmium in the clam *Ruditapes philippinarum* using combined ionomic, metabolomic and transcriptomic analyses. Environ. Pollut..

[13] Li Y.Q., Chen C.M., Liu N., Wang L. (2021). Cadmium-induced ultrastructural changes and apoptosis in the gill of freshwater mussel *Anodonta woodiana*. Environ. Sci. Pollut. Res..

[14] Wang X., Li P., Xie H., Wang X., Zhang Q., Ding X., Zhang W., Zhang L., Moshiree B., Espinosa A. (2022). Recent patents relating to employing metabolomic data to diagnose disease states and determine the likelihood that a patient will respond to certain treatments. Nat. Biotechnol..

[15] Chandhini S., Kumar V.J.R. (2018). Transcriptomics in aquaculture: Current status and applications. Rev. Aquac..

[16] Liu H., Li H., Zhang X., Gong X., Han D., Zhang H., Tian X., Xu Y. (2021). Metabolomics comparison of metabolites and functional pathways in the gills of *Chlamys farreri* under cadmium exposure. Environ. Toxicol. Pharmacol..

[17] Chen X., Liu H., Huang H., Liber K., Jiang T., Yang J. (2021). Cadmium bioaccumulation and distribution in the freshwater bivalve *Anodonta woodiana* exposed to environmentally relevant Cd levels. Sci. Total. Environ..

[18] Wang J., Deng W., Zou T., Bai B., Chang A.K., Ying X. (2021). Cadmium-induced oxidative stress in *Meretrix meretrix* gills leads to mitochondria-mediated apoptosis. Ecotoxicology.

[19] Wadige C.P.M.M., Taylor A.M., Krikowa F., Lintermans M., Maher W.A. (2017). Exposure of the freshwater bivalve *Hyridella australis* to metal contaminated sediments in the field and laboratory microcosms: Metal uptake and effects. Ecotoxicology.

[20] Guo Y., Lei Y., Xu W., Zhang Y., Zhou H., Zhang W., Mai K. (2018). Protective effects of dietary selenium on Abalone *Haliotis discus* hannai against the toxicity of waterborne cadmium. Aquac. Res..

[21] Zhang X., Zhang W., Zhao L., Zheng L., Wang B., Song C., Liu S. (2024). Mechanisms of Gills Response to Cadmium Exposure in Greenfin Horse-Faced Filefish (*Thamnaconus septentrionalis*): Oxidative Stress, Immune Response, and Energy Metabolism. Animals.

[22] Liu X., Li Z., Li Q., Bao X., Jiang L., Yang J. (2024). Acute exposure to *Polystyrene nanoplastics* induced oxidative stress in *Sepia esculenta* Larvae. Aquac. Rep..

[23] Wang Y., Chen X., Xu X., Yang J., Liu X., Sun G., Li Z. (2023). Weighted Gene Co-Expression Network Analysis Based on Stimulation by Lipopolysaccharides and Polyinosinic:polycytidylic Acid Provides a Core Set of Genes for Understanding Hemolymph Immune Response Mechanisms of *Amphioctopus fangsiao*. Animals.

[24] Wang Y., Liu X., Wang W., Sun G., Feng Y., Xu X., Li B., Luo Q., Li Y., Yang J. (2024). The Investigation on Stress Mechanisms of *Sepia esculenta* Larvae in the Context of Global Warming and Ocean Acidification. Aquac. Rep..

[25] Alfaro A.C., Young T. (2016). Showcasing Metabolomic Applications in Aquaculture: A Review. Rev. Aquacult..

[26] Castaldo G., Delahaut V., Slootmaekers B., Bervoets L., Town R.M., Blust R., De Boeck G. (2021). A comparative study on the effects of three different metals (Cu, Zn and Cd) at similar toxicity levels in common carp, *Cyprinus carpio*. J. Appl. Toxicol..

[27] Fu S., Lu Y., Zhang X., Yang G., Chao D., Wang Z., Shi M., Chen J., Chao D.-Y., Li R. (2019). The ABC transporter ABCG36 is required for cadmium tolerance in rice. J. Exp. Bot..

[28] Espinoza H.M., Williams C.R., Gallagher E.P. (2011). Effect of cadmium on glutathione S-transferase and metallothionein gene expression in *Coho salmon* liver, gill and olfactory tissues. Aquat. Toxicol..

[29] Hemelraad J., Holwerda D.A., Herwig H.J., Zandee D.I. (1990). Effects of cadmium in freshwater clams. III. Interaction with energy metabolism in *Anodonta cygnea*. Arch. Environ. Contam. Toxicol..

[30] Zhan J., Sun T., Wang X., Wu H., Yu J. (2023). Meta-analysis reveals the species-, dose- and duration-dependent effects of cadmium toxicities in marine bivalves. Sci. Total. Environ..

[31] Kim H., Yim B., Kim J., Kim H., Lee Y.-M. (2017). Molecular Characterization of ABC Transporters in Marine Ciliate, *Euplotes Crassus*: Identification and Response to Cadmium and Benzo[α]Pyrene. Mar. Pollut. Bull..

[32] Lerebours A., To V.V., Bourdineaud J.-P. (2016). *Danio Rerio* ABC Transporter Genes Abcb3 and Abcb7 Play a Protecting Role against Metal Contamination. J. Appl. Toxicol..

[33] Guo B., Xu Z., Yan X., Buttino I., Li J., Zhou C., Qi P. (2020). Novel ABCB1 and ABCC Transporters Are Involved in the Detoxification of Benzo(α)Pyrene in Thick Shell Mussel, *Mytilus coruscus*. Front. Mar. Sci..

[34] Lv J.-J., Yuan K.-K., Lu G.-X., Li H.-Y., Kwok H.F., Yang W.-D. (2023). Responses of ABCB and ABCC transporters to the toxic dinoflagellate *Prorocentrum lima* in the mussel *Perna viridis*. Aquat. Toxicol..

[35] Bieczynski F., Painefilú J.C., Venturino A., Luquet C.M. (2021). Expression and Function of ABC Proteins in Fish Intestine. Front. Physiol..

[36] Oezen G., Schentarra E.-M., Bolten J.S., Huwyler J., Fricker G. (2022). Sodium arsenite but not aluminum chloride stimulates ABC transporter activity in renal proximal tubules of killifish (*Fundulus heteroclitus*). Aquat. Toxicol..

[37] Tian J., Hu J., Liu G., Yin H., Chen M., Miao P., Bai P., Yin J. (2018). Altered Gene expression of ABC transporters, nuclear receptors and oxidative stress signaling in zebrafish embryos exposed to CdTe quantum dots. Environ. Pollut..

[38] Lubyaga Y., Yarinich L., Drozdova P., Pindyurin A., Gurkov A., Luckenbach T., Timofeyev M. (2023). The ABCs of the Amphipod P-Glycoprotein: Heterologous Production of the Abcb1 Protein of a Model Species *Eulimnogammarus verrucosus* (Amphipoda: Gammaridae) from Lake Baikal. Comp. Biochem. Physiol. C Toxicol. Pharmacol..

[39] Luo S.-S., Chen X.-L., Wang A.-J., Liu Q.-Y., Peng M., Yang C.-L., Yin C.-C., Zhu W.-L., Zeng D.-G., Zhang B. (2024). Genome-wide analysis of ATP-binding cassette (ABC) transporter in *Penaeus vannamei* and identification of two ABC genes involved in immune defense against *Vibrio parahaemolyticus* by affecting NF-κB signaling pathway. Int. J. Biol. Macromol..

[40] Wang H., Liu Y., Peng Z., Li J., Huang W., Liu Y., Wang X., Xie S., Sun L., Han E. (2019). Ectopic Expression of Poplar ABC Transporter PtoABCG36 Confers Cd Tolerance in *Arabidopsis thaliana*. Int. J. Mol. Sci..

[41] Zeng Y., Charkowski A.O. (2021). The Role of ATP-Binding Cassette Transporters in Bacterial Phytopathogenesis. Phytopathology®.

[42] Thévenod F., Lee W.-K. (2024). Cadmium transport by mammalian ATP-binding cassette transporters. BioMetals.

[43] Esquivel B.D., Rybak J.M., Barker K.S., Fortwendel J.R., Rogers P.D., White T.C. (2020). Characterization of the Efflux Capability and Substrate Specificity of *Aspergillus fumigatus* PDR5-like ABC Transporters Expressed in *Saccharomyces cerevisiae*. mBio.

[44] Kropf C., Segner H., Fent K. (2016). ABC Transporters and Xenobiotic Defense Systems in Early Life Stages of Rainbow Trout (*Oncorhynchus mykiss*). Comp. Biochem. Physiol. C Toxicol. Pharmacol..

[45] Wang H., Liu S., Xun X., Li M., Lou J., Zhang Y., Shi J., Hu J., Bao Z., Hu X. (2020). Toxin- and species-dependent regulation of ATP-binding cassette (ABC) transporters in scallops after exposure to paralytic shellfish toxin-producing dinoflagellates. Aquat. Toxicol..

[46] Brunetti P., Zanella L., De Paolis A., Di Litta D., Cecchetti V., Falasca G., Barbieri M., Altamura M.M., Costantino P., Cardarelli M. (2015). Cadmium-inducible expression of the ABC-type transporter AtABCC3 increases phytochelatin-mediated cadmium tolerance in Arabidopsis. J. Exp. Bot..

[47] Zhang X.D., Zhao K.X., Yang Z.M. (2018). Identification of Genomic ATP Binding Cassette (ABC) Transporter Genes and Cd-Responsive ABCs in *Brassica napus*. Gene.

[48] Kumari S., Kumar M., Gaur N.A., Prasad R. (2021). Multiple roles of ABC transporters in yeast. Fungal Genet. Biol..

[49] Ren J., Liu S., Zhang Q., Zhang Z., Shang S. (2024). Effects of Cadmium Exposure on Haemocyte Immune Function of Clam *Ruditapes philippinarum* at Different Temperatures. Mar. Environ. Res..

[50] Della Torre C., Bocci E., Focardi S.E., Corsi I. (2014). Differential ABCB and ABCC gene expression and efflux activities in gills and hemocytes of *Mytilus galloprovincialis* and their involvement in cadmium response. Mar. Environ. Res..

[51] Pan C., Lu H., Yu J., Liu J., Liu Y., Yan C. (2019). Identification of Cadmium-responsive *Kandelia obovata* SOD family genes and response to Cd toxicity. Environ. Exp. Bot..

[52] Cheng C., Ma H., Liu G., Fan S., Guo Z. (2022). Mechanism of Cadmium Exposure Induced Hepatotoxicity in the Mud Crab (*Scylla paramamosain*): Activation of Oxidative Stress and Nrf2 Signaling Pathway. Antioxidants.

[53] Ferreira M.J., Rodrigues T.A., Pedrosa A.G., Silva A.R., Vilarinho B.G., Francisco T., Azevedo J.E. (2023). Glutathione and peroxisome redox homeostasis. Redox Biol..

[54] Mirkovic J.J., Kocic G., Jurinjak Z., Alexopoulos C. (2022). Protective role of glutathione in oxidative stress caused by cadmium and copper. Eur. Psychiatry.

[55] Berndt C., Lillig C.H. (2017). Glutathione, Glutaredoxins, and Iron. Antioxid. Redox Signal..

[56] Eroglu A., Dogan Z., Kanak E.G., Atli G., Canli M. (2014). Effects of heavy metals (Cd, Cu, Cr, Pb, Zn) on fish glutathione metabolism. Environ. Sci. Pollut. Res..

[57] da Souza I.C., Morozesk M., Bonomo M.M., Azevedo V.C., Sakuragui M.M., Elliott M., Matsumoto S.T., Wunderlin D.A., Baroni M.V., Monferrán M.V. (2018). Differential Biochemical Responses to Metal/Metalloid Accumulation in Organs of an Edible Fish (*Centropomus parallelus*) from Neotropical Estuaries. Ecotoxicol. Environ. Saf..

[58] Flohé L., Toppo S., Orian L. (2022). The glutathione peroxidase family: Discoveries and mechanism. Free. Radic. Biol. Med..

[59] Chrestensen C.A., Starke D.W., Mieyal J.J. (2000). Acute Cadmium Exposure Inactivates Thioltransferase (Glutaredoxin), Inhibits Intracellular Reduction of Protein-glutathionyl-mixed Disulfides, and Initiates Apoptosis. J. Biol. Chem..

[60] Bachhawat A.K., Yadav S. (2018). The glutathione cycle: Glutathione metabolism beyond the γ-glutamyl cycle. Iubmb Life.

[61] Das U., Rahman M.A., Ela E.J., Lee K.-W., Kabir A.H. (2020). Sulfur Triggers Glutathione and Phytochelatin Accumulation Causing Excess Cd Bound to the Cell Wall of Roots in Alleviating Cd-Toxicity in Alfalfa. Chemosphere.

[62] Monteiro M., Rimoldi S., Costa R.S., Kousoulaki K., Hasan I., Valente L.M.P., Terova G. (2023). Polychaete (*Alitta virens*) meal inclusion as a dietary strategy for modulating gut microbiota of European seabass (*Dicentrarchus labrax*). Front. Immunol..

[63] Kotera M., Bayashi T., Hattori M., Tokimatsu T., Goto S., Mihara H., Kanehisa M. (2010). Comprehensive Genomic Analysis of Sulfur-Relay Pathway Genes. Genome Inform. Int. Conf. Genome Inform..

[64] Macias-Barragan J., Huerta-Olvera S.G., Hernandez-Cañaveral I., Pereira-Suarez A.L., Montoya-Buelna M. (2017). Cadmium and α-lipoic acid activate similar de novo synthesis and recycling pathways for glutathione balance. Environ. Toxicol. Pharmacol..

[65] Rigoulet M., Bouchez C., Paumard P., Ransac S., Cuvellier S., Duvezin-Caubet S., Mazat J., Devin A. (2020). Cell energy metabolism: An update. Biochim. Biophys. Acta BBA Bioenerg..

[66] Jin P., Zhang J., Wan J., Overmans S., Gao G., Ye M., Dai X., Zhao J., Xiao M., Xia J. (2021). The Combined Effects of Ocean Acidification and Heavy Metals on Marine Organisms: A Meta-Analysis. Front. Mar. Sci..

[67] Chang Y.-C., Kim C.-H. (2022). Molecular Research of Glycolysis. Int. J. Mol. Sci..

[68] Ramírez-Bajo M.J., de Atauri P., Ortega F., Westerhoff H.V., Gelpí J.L., Centelles J.J., Cascante M. (2014). Effects of Cadmium and Mercury on the Upper Part of Skeletal Muscle Glycolysis in Mice. PLoS ONE.

[69] Dong A., Huo J., Yan J., Dong A. (2021). Oxidative stress in liver of turtle *Mauremys reevesii* caused by cadmium. Environ. Sci. Pollut. Res..

[70] Hanana H., Kleinert C., André C., Gagné F. (2018). Influence of Cadmium on Oxidative Stress and NADH Oscillations in Mussel Mitochondria. Comp. Biochem. Physiol. C Toxicol. Pharmacol..

[71] Lee J.-W., Jo A.-H., Lee D.-C., Choi C.Y., Kang J.-C., Kim J.-H. (2023). Review of cadmium toxicity effects on fish: Oxidative stress and immune responses. Environ. Res..

[72] Zhang Z., Zheng Z., Cai J., Liu Q., Yang J., Gong Y., Wu M., Shen Q., Xu S. (2017). Effect of cadmium on oxidative stress and immune function of common carp (*Cyprinus Carpio* L.) by transcriptome analysis. Aquat. Toxicol..

[73] Faverney C.R.-D., Orsini N., de Sousa G., Rahmani R. (2004). Cadmium-induced apoptosis through the mitochondrial pathway in rainbow trout hepatocytes: Involvement of oxidative stress. Aquat. Toxicol..

[74] Shekh K., Tang S., Kodzhahinchev V., Niyogi S., Hecker M. (2019). Species and life-stage specific differences in cadmium accumulation and cadmium induced oxidative stress, metallothionein and heat shock protein responses in white sturgeon and rainbow trout. Sci. Total. Environ..

[75] MacLean A., Legendre F., Appanna V.D. (2023). The tricarboxylic acid (TCA) cycle: A malleable metabolic network to counter cellular stress. Crit. Rev. Biochem. Mol. Biol..

[76] Lu Z., Wang S., Ji C., Li F., Cong M., Shan X., Wu H. (2020). iTRAQ-based proteomic analysis on the mitochondrial responses in gill tissues of juvenile olive flounder *Paralichthys olivaceus* exposed to cadmium. Environ. Pollut..

[77] Kabir A., Rabbane G., Hernandez M.R., Shaikh A.A., Moniruzzaman M., Chang X. (2024). Impaired intestinal immunity and microbial diversity in common carp exposed to cadmium. Comp. Biochem. Physiol. Part C Toxicol. Pharmacol..

[78] Matić D., Vlahović M., Ilijin L., Grčić A., Filipović A., Todorović D., Perić-Mataruga V. (2021). Implications of long-term exposure of a *Lymantria dispar* L. population to pollution for the response of larval midgut proteases and acid phosphatases to chronic cadmium treatment. Comp. Biochem. Physiol. Part C Toxicol. Pharmacol..

