# Supplementary material for: Exploration of Response Mechanisms in the Gills of Pacific Oyster (Crassostrea gigas) to Cadmium Exposure through Integrative Metabolomic and Transcriptomic Analyses
Source: Animals (Basel). 2024 Aug 9;14(16):2318. doi: 10.3390/ani14162318 (PMC11350665; doi:10.3390/ani14162318)
Supplement: Supplementary file 1 [file animals-14-02318-s001.zip › FIgure S1.pdf]

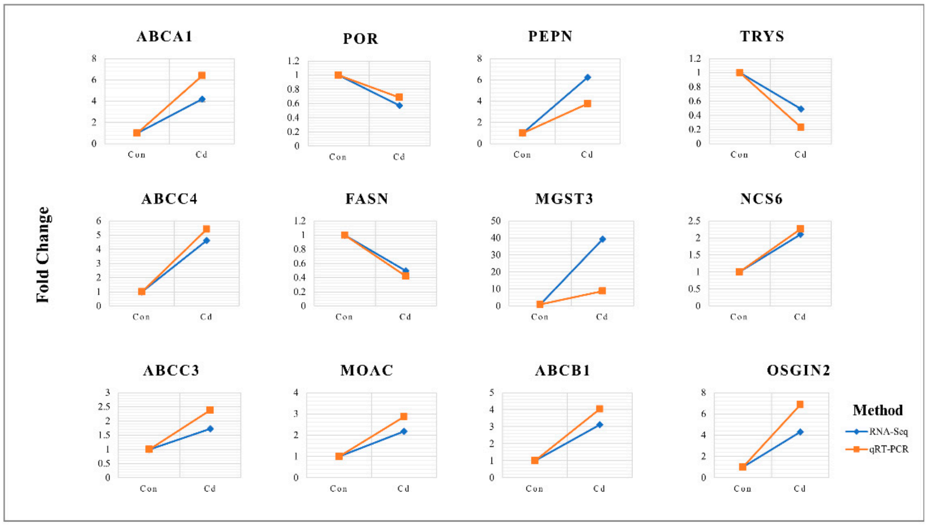

Figure S1. Comparison of qPCR and RNA-Seq results of 12 hub genes. Expression levels of key genes were standardized using the expression levels of β-actin genes. The x-axis represents the Cd-exposed group and the control group, while the y-axis represents the fold change in gene expression comparing the Cd-exposed group to the control group. The letters indicate the level of significant difference between groups ($P < 0.05$).
